# Ovarian cycle stage critically affects 21-gene recurrence scores in *Mmtv-Pymt* mouse mammary tumours

**DOI:** 10.1186/s12885-021-08496-y

**Published:** 2021-06-26

**Authors:** Sarah M. Bernhardt, Pallave Dasari, Danielle J. Glynn, Lucy Woolford, Lachlan M. Moldenhauer, David Walsh, Amanda R. Townsend, Timothy J. Price, Wendy V. Ingman

**Affiliations:** 1 Discipline of Surgery, Adelaide Medical School, University of Adelaide, The Queen Elizabeth Hospital, Adelaide, 28 Woodville Rd, Woodville, 5011 Australia; 2 Robinson Research Institute, University of Adelaide, Adelaide, Australia; 3 School of Animal and Veterinary Sciences, University of Adelaide, Adelaide, SA Australia; 4 Adelaide Medical School, University of Adelaide, Adelaide, Australia; 5 Department of Medical Oncology, The Queen Elizabeth Hospital, Adelaide, SA Australia

**Keywords:** Premenopausal Breast cancer, Predictive biomarkers, Ovarian cycle, Genomics, Mouse models

## Abstract

**Background:**

The Oncotype DX 21-gene Recurrence Score is predictive of adjuvant chemotherapy benefit for women with early-stage, estrogen receptor (ER)-positive, HER2-negative breast cancer. In premenopausal women, fluctuations in estrogen and progesterone during the menstrual cycle impact gene expression in hormone-responsive cancers. However, the extent to which menstrual cycling affects the Oncotype DX 21-gene signature remains unclear. Here, we investigate the impact of ovarian cycle stage on the 21-gene signature using a naturally cycling mouse model of breast cancer.

**Methods:**

ER-positive mammary tumours were dissected from naturally cycling *Mmtv-Pymt* mice at either the estrus or diestrus phase of the ovarian cycle. The Oncotype DX 21-gene signature was assessed through quantitative real time-PCR, and a 21-gene experimental recurrence score analogous to the Oncotype DX Recurrence Score was calculated.

**Results:**

Tumours collected at diestrus exhibited significant differences in expression of 6 Oncotype DX signature genes (*Ki67*, *Ccnb1*, *Esr1*, *Erbb2*, *Grb7*, *Bag1*; *p* ≤ 0.05) and a significant increase in 21-gene recurrence score (21.8 ± 2.4; mean ± SEM) compared to tumours dissected at estrus (15.5 ± 1.9; *p* = 0.03). Clustering analysis revealed a subgroup of tumours collected at diestrus characterised by increased expression of proliferation- (*p* < 0.001) and invasion-group (*p* = 0.01) genes, and increased 21-gene recurrence score (*p* = 0.01). No correlation between ER, PR, HER2, and KI67 protein abundance measured by Western blot and abundance of mRNA for the corresponding gene was observed, suggesting that gene expression is more susceptible to hormone-induced fluctuation compared to protein expression.

**Conclusions:**

Ovarian cycle stage at the time of tissue collection critically affects the 21-gene signature in *Mmtv-Pymt* murine mammary tumours. Further studies are required to determine whether Oncotype DX Recurrence Scores in women are similarly affected by menstrual cycle stage.

**Supplementary Information:**

The online version contains supplementary material available at 10.1186/s12885-021-08496-y.

## Introduction

The Oncotype DX 21-gene Recurrence Score assay is used in clinical practice for guiding adjuvant chemotherapy treatment decisions for women with early-stage, estrogen receptor (ER)-positive, HER2-negative breast cancer [1–4]. The Oncotype DX assay quantifies the expression of 16 genes associated with proliferation (*MKI67*, *STK15*, *BIRC5*, *CCNB1*, *MYBL2*), estrogen signalling (*ESR1*, *PGR*, *BCL2*, *SCUBE2*), HER2 (*ERBB2*, *GRB7*), invasion (*MMP11*, *CSTV*) and other genes (*GSTM1*, *BAG1*, *CD68*), as well as 5 house-keeping genes. These 21 signature genes are combined in an algorithm to calculate a Recurrence Score that reflects the patient’s underlying risk of disease recurrence and predicts the likely benefit from the addition of chemotherapy to endocrine treatment [5–10]. The Oncotype DX assay guides chemotherapy treatment decisions for both premenopausal and postmenopausal breast cancer patients [11], however it was developed predominantly in postmenopausal women [12]. Differences in chemotherapy benefit between young women with intermediate Recurrence Scores compared to older women has recently been described [10]. Moreover, a recent study has demonstrated that results generated by the Oncotype DX 21-gene algorithm are affected by patient age at the time of tissue collection [13]. Together, these observations suggest that the algorithm is affected by underlying differences in tumour biology in premenopausal women that are yet to be defined.

Menstrual cycling in younger premenopausal women could affect the accuracy of the Oncotype DX algorithm. In premenopausal women, ER-positive breast cancers are highly responsive to fluctuations in ovarian hormones that occur across the menstrual cycle. Breast cancer biopsies collected from women during the luteal phase of the menstrual cycle exhibit increased proliferation compared to paired biopsies collected during the follicular phase [14]. Similarly, growth factor receptor expression is increased at the luteal phase of the menstrual cycle, when circulating concentrations of progesterone peak [15]. Expression of a number of Oncotype DX signature genes also varies with menstrual cycle stage, including the estrogen-regulated *PGR* gene and proliferation-associated genes *MKI67*, *CCNB1*, *BIRC5,* and *MYBL2* [16–18]. Evidence that hormone-induced gene expression changes could impact the Oncotype DX gene signature comes from in vitro findings that estrogen and progesterone co-treatment of a breast cancer cell line increases Oncotype DX Recurrence Score compared to estrogen treatment alone [19]. However, despite the known effects of ovarian hormones on breast cancer gene expression, whether menstrual cycling in premenopausal breast cancer patients affects the Oncotype DX Recurrence Score has not been directly addressed. A key limitation in advancing this issue is the lack of availability of large human breast cancer datasets with matched clinical information on menstrual cycle stage [20]. To partially address this limitation, animal models can provide early evidence to precede large-scale clinical studies.

Transgenic mice harbouring the polyomavirus middle T-antigen oncogene driven by the mammary gland specific mouse mammary tumour virus promoter (*Mmtv-Pymt*) exhibit well-defined histopathological features and biomarkers with direct relevance to Oncotype DX. In *Mmtv-Pymt* mice, tumour progression to malignancy occurs in a number of stages that are comparable to human disease progression from in situ lesions to invasive carcinoma [21]. *Mmtv-Pymt* tumours express ER and PR in the earlier stages of tumour development and are responsive to endocrine treatment [21, 22]. As tumours progress to become more aggressive and metastatic, *Erbb2* and *Ccnd1* increase in expression [21]. Biomarkers *Mki67* and *Bcl2* are also associated with tumour progression in the *Mmtv-Pymt* model [23]. Use of the *Mmtv-Pymt* model in pre-clinical studies was instrumental in establishing the role of macrophages in breast cancer progression [24], with macrophage-specific CD68 now recognised as a promising prognostic and predictive biomarker [25, 26] which is included in the Oncotype DX signature. Thus, *Mmtv-Pymt* transgenic mice are an ideal model to investigate the impact of ovarian cycling on Oncotype DX Recurrence Scores as mammary tumours express many Oncotype DX signature genes that have been demonstrated to be associated with disease progression.

In this study, we investigate the impact of ovarian cycle stage on the Oncotype DX 21-gene signature in mammary tumours from naturally cycling *Mmtv-Pymt* mice. Mammary tumours were dissected from *Mmtv-Pymt* transgenic mice at either the estrus or diestrus phase of the ovarian cycle. These two phases of the cycle were used as estrus phase has been shown previously to exhibit high estradiol and low progesterone and diestrus phase exhibits moderate estradiol and high progesterone, comparable to the follicular and luteal phase of the human menstrual cycle respectively [27, 28]. ER-positivity was confirmed by immunohistochemistry. The Oncotype DX 21-gene signature was assessed through quantitative real time-PCR, and 21-gene experimental recurrence scores analogous to the Oncotype DX platform were calculated [5]. Results from this study show that ovarian cycle stage affects expression of the 21-gene signature and experimental recurrence scores in *Mmtv-Pymt* mammary tumours. Mammary tumours dissected from mice at diestrus exhibit increased expression of proliferative and HER2-associated genes, and increased 21-gene experimental recurrence scores, compared to tumours dissected at estrus. Furthermore, clustering analysis identified a subset of tumours collected at diestrus that may be more sensitive to hormonal fluctuations during the ovarian cycle, and more prone to cycle-induced changes in recurrence scores. This study provides pre-clinical evidence that menstrual cycling might affect Oncotype DX Recurrence Scores in premenopausal women.

## Methods

### *Mmtv-Pymt* transgenic mice

Ethics approval was obtained from The University of Adelaide Animal Ethics Committee (Approval number M2016–124) and all experiments were performed in accordance with the Australian Code of Practice for the Care and use of Animals for Scientific Purposes. All mice were maintained in pathogen-free conditions with 12:12 light-dark cycles and controlled temperature. All methods are reported in accordance with ARRIVE guidelines (https://arriveguidelines.org).

Transgenic mice harbouring the polyomavirus middle T-antigen oncogene (*Pymt*) driven by the mammary gland specific mouse mammary tumour virus promoter (*Mmtv*; *Mmtv-Pymt*) were used to model the effect of ovarian cycle stage on the 21-gene signature. Tumours from *Mmtv-Pymt* mice are ER-positive in the early stage of development and progress to become ER-negative [21]. Immunohistochemistry were performed to confirm that tumours were estrogen receptor (ER)-positive.

For inclusion in the study it was required that mice exhibited normal estrous cycles, defined as 4 to 5 days between one estrus phase and the next estrus phase. To assess this, once tumours were palpable estrous cycles were tracked by daily vaginal smearing for at least 2 complete cycles prior to tissue collection, as previously described [28, 29]. Mice were randomly assigned to be euthanised at either the estrus or diestrus phase of the estrous cycle.

Mice were euthanised by cardiac puncture under deep anaesthesia (500 μl of 2% Avertin (Sigma-Aldrich) injected intraperitoneally) followed by cervical dislocation at either the estrus or diestrus stage of the estrous cycle, and mammary tumours dissected. The primary tumour was cut in half. Half the tumour was fixed in 4% paraformaldehyde overnight at 4 °C, washed thrice with 1 × PBS, and stored in 70% ethanol prior to processing and embedding in paraffin wax. The other half of the tumour was immediately snap-frozen in liquid nitrogen and stored at − 80 °C until RNA extraction was performed.

### Haematoxylin eosin staining

Haematoxylin and eosin (H&E) staining was performed on 5uM paraffin-embedded sections. Sections were dewaxed in xylene, passed sequentially through 100, 90, 70 and 50% ethanol for rehydration. The sections were then stained with haematoxylin and counterstained with eosin, prior to dehydrating and mounting with Entellan mounting medium. H&E stains of primary mammary tumours dissected from *Mmtv-Pymt* mice were assessed by a veterinary pathologist blind to mouse estrous cycle phase, to confirm that tumour tissue were of mammary epithelial cell origin, and to grade tumours.

### Microscopy

Tissue sections stained by immunohistochemistry or H&E were captured as a digital image using a Nanozoomer 1.0 (Hamamatsu, Shizouka, Japan) digital imager at a zoom equivalent to a 40 x objective lens. All microscopy images are shown as they were captured, and have not been adjusted (ie brightness/contrast, colour, resolution, processing, averaging or sharpen/soften) in any way.

### RNA extraction and complementary DNA (cDNA) synthesis

Total RNA was extracted from snap-frozen mammary tumours using TRIzol (Life technologies). Samples were treated with TURBO DNA-free DNase kit (Life Technologies) to remove genomic DNA contamination. cDNA was reverse transcribed from 500 ng of RNA using iScript cDNA synthesis kit (Bio-Rad), with reactions incubated at 25 °C for 5 min, 42 °C for 25 min, and 85 °C for 5 min.

### Quantitative real time-PCR

Real time-PCR amplification was performed on a Bio-Rad CFX384 Real-Time Detection System using SYBR Green PCR Master Mix (Bio-Rad). Reactions were loaded into 384-well plates in triplicate, using a QIAgility robot (Qiagen). PCR conditions were 95 °C for 10 min, then 50 cycles of 95 °C for 15 s, 60 °C for 15 s, 72 °C for 30 s.

The 21-gene signature was assessed through quantitative real time-PCR. Primer pairs are detailed in Table 1. A 21-gene experimental recurrence score analogous to the Oncotype DX Recurrence Score was calculated using the Oncotype DX Recurrence Score algorithm.

**Table 1:** PCR primers used to quantify gene expression in Mmtv-Pymt murine mammary tumours. Primers recognise mouse genes

Gene	Primer sequence	Product length (bp)
Mki67	5' – AATCCAACTCAAGTAAACGGGG 3' - TTGGCTTGCTTCCATCCTCA	127
Stk15	5' – CTGGATGCTGCAAACGGATAG 3' - CGAAGGGAACAGTGGTCTTAACA	105
Birc5	5' – CTACCGAGAACGAGCCTGATT 3' - AGCCTTCCAATTCCTTAAAGCAG	60
Ccnb1	5' – AGAGCTATCCTCATTGACTGGC 3' - AACATGGCCGTTACACCGAC	155
Mybl2	5' – GTGAGGCAGTTTGGACAGCAA 3' - GGATTCAAAACCCTCAGCCA	101
Esr1	5' – TGATTGGTCTCGTCTGGCGCT 3' - GCACACAAACTCTTCTCCCTGC	179
Pgr	5' – CGCCATCTACCAGCCGCTC 3' - TGAATCTGGCCTCAGGTAGTT	121
Bcl2	5' – ATGCCTTTGTGGAACTATATGGC 3' - GGTATGCACCCAGAGTGATGC	120
Scube2	5' – GGCTGTGTCCACGACTGTTTA 3' - GTTCTCCAAGCATTCGTCCAT	117
Erbb2	5' – TGCTCAACTGGTGTGTTCAGATT 3' - TTCGGGCAGCTAGGTCC	84
Grb7	5' – ACAAACAGGCATATCCCATGAAG 3' - TAGAGGCCAGATCGACGCA	159
Mmp11	5' – GCCTGATGTACTGAATGCCC 3' - GCTCCCTTACAAGCTGCCA	119
Ctsv	5' – ATCAAACCTTTAGTGCAGAGTGG 3' - CTGTATTCCCCGTTGTGTAGC	136
Gstm1	5' – ATACTGGGATACTGGAACGTCC 3' - AGTCAGGGTTGTAACAGAGCAT	349
Cd68	5' – TGTCTGATCTTGCTAGGACCG 3' - GAGAGTAACGGCCTTTTTGTGA	75
Bag1	5' – GCAGCAGGGAGTTGACTAGAA 3' - TTACTTCCTCGGTTTGGGTCG	111
Actb	5' – GTGTGACGTTGACATCCGTAAAG 3' - CTCAGGAGGAGCAATGATCTTGAT	151
Gapdh	5' – ACACATTGGGGGTAGGAACA 3' - AACTTTGGCATTGTGGAAGG	223
Rplp0	5' – AGATTCGGGATATGCTGTTGGC 3' - TCGGGTCCTAGACCAGTGTTC	109
Gusb	5' – CGGGCTGGTGACCTACTGGATT 3' - TGGCACTGGGAACCTGAAGT	134
Tfrc	5' – GTTTCTGCCAGCCCCTTATTAT 3' - GCAAGGAAAGGATATGCAGCA	152

### Calculation of 21-gene experimental recurrence scores

Normalised gene expression measurements were calculated as Δ CT = CT (mean of five reference genes) – CT (gene of interest) + 10. A 1-unit increase in reference-normalised expression measurements reflects a doubling of RNA. Experimental recurrence scores were calculated from reference-normalised gene expression, using the Oncotype DX 21-gene Recurrence Score algorithm, as previously described [13].

### Protein extraction and Western blot analysis

Total protein was extracted from snap-frozen mammary tumours using Triton X-100 lysis buffer (50 mM Tris pH 8.0, 150 mM sodium chloride, 1% Triton X-100, protease inhibitor cocktail (Roche)). Samples were quantified using Bradford Protein Assay (ThermoFisher), and 20 μg of protein was separated on a 12% precast polyacrylamide gel (BioRad) at 30 mA for 40 min. Proteins were transferred onto a nitrocellulose membrane using Trans-Blot Turbo Transfer Packs (BioRad) in a Trans-Blot Turbo Transfer chamber system (BioRad). Membranes were blocked with 5% w/v skim milk in TBS-T (50 mM Tris-Cl pH 7.5, 50 mM NaCl, 0.2% Tween-20) for 1 h at room temperature, prior to incubation with primary antibodies for 1 h at room temperature. Primary antibodies were anti-ER (Santa Cruz, 1:500), anti-PR (Santa Cruz, 1:500), anti-HER2 (Cell Signalling Technologies, 1:500), anti-Ki67 (Abcam, 1:500) and anti-βactin (Abcam, 1:1000). Membranes were then washed thrice with TBS-T, prior to incubation with appropriate anti-mouse (Dako, 1:2000) or anti-rabbit (Dako, 1:2000) antibodies for 30 min at room temperature. Membranes were exposed using enhanced chemiluminescence (ECL; BioRad) and visualised using a Fuji LAS4000 Luminescent Image Analyser. To re-probe, membranes were first stripped using a low pH glycine stripping buffer (1.5% glycine pH 2.2, 0.1% SDS, 1% Tween) for 10 min at room temperature, prior to re-blocking and subsequent re-probing with primary antibodies as described above. All western blot images are shown as they were imaged, and have not been adjusted (ie brightness/contrast, colour, resolution, processing, averaging or sharpen/soften) in any way.

### Statistical analyses

All data were assessed using SPSS Statistics Version 24 (IBM Corporation, Armonk, NY, USA) or SAS 9.4 (SAS Institute Inc., Cary, NC, USA). Gene expression and 21-gene experimental recurrence scores were compared between ovarian cycle stages using independent T-tests. Differences in gene expression and 21-gene experimental recurrence scores between clusters were assessed using one-way analysis of variance with post hoc comparisons performed. Correlation between mRNA and protein expression was assessed using Spearman’s rank correlation coefficient. Data were considered significant when *p* ≤ 0.05.

## Results

### Assessment of tumour clinical features for inclusion in study

**Fig. 1:**
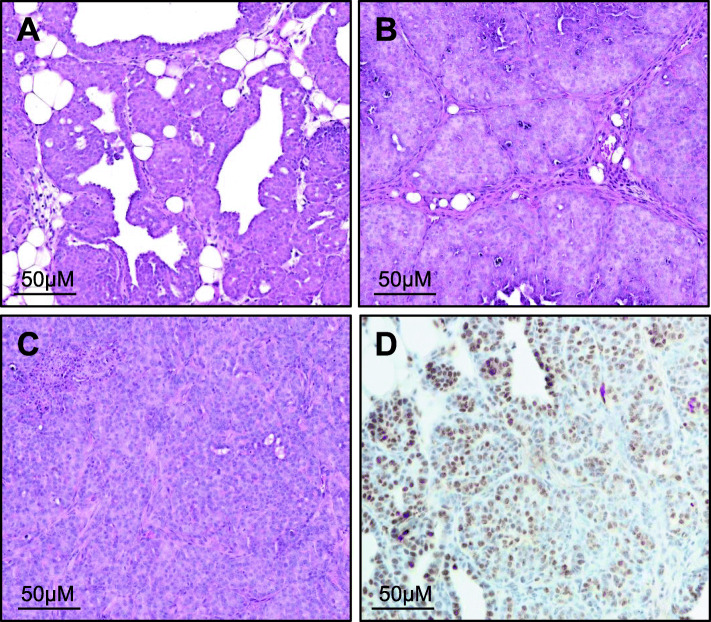
Histological and immunohistochemical analysis of *Mmtv-Pymt* mammary tumours. Representative images of haematoxylin and eosin stained *Mmtv-Pymt* mammary tumours of different grades. Tumours were assessed and graded by a veterinary pathologist. **A** grade 2, **B** grade 3, and **C** grade 4 tumours. **D** Representative images of estrogen receptor staining in *Mmtv-Pymt* mammary tumours. Bars represent 50 μM

Hematoxylin and eosin stains were performed on primary tumours collected from a total of 60 mice, and tumours were assessed and graded by a veterinary pathologist. Tumours were included in the study if tumour tissue were of mammary epithelial cell origin. Examples of hematoxylin and eosin stained mammary tumours are presented in Fig. 1A-C and Supplementary figures 1, 2 and 3. Mammary tumours were also assessed for ER positivity through immunohistochemistry. All tumours stained positive for ER. An example of ER staining is presented in Fig. 1D and Supplementary figure 4.

Of the 60 mice in the study, 1 mouse was excluded from analysis as the tumour was salivary gland epithelial cell in origin. Two mice were excluded from analysis as smearing was performed for less than 2 full cycles. These mice were culled for ethical reasons before 2 cycles could be tracked, as the primary tumour size exceeded the maximum 2000mm^3^ tumour burden threshold. A further 4 mice were excluded from analysis as they were not cycling normally. A total of 53 mice were included in analysis (estrus *n* = 25; diestrus *n* = 28). Tumour and mouse characteristics are presented in Table 2.

**Table 2:** Clinical features of primary mammary tumours collected from *Mmtv-Pymt* mice that met the inclusion criteria for the study. Mammary tumours were collected from *Mmtv-Pymt* transgenic mice at either estrus or diestrus phase of the ovarian cycle. Ovarian cycle stage was determined by cytological analysis of vaginal smears, performed for at least 2 weeks. All tumours are estrogen receptor positive as determined through immunohistochemistry. No differences in tumour features were observed between ovarian cycle stages

Characteristics	Estrus n (%)	Diestrus n (%)
**Total Number**	25	28
**Mouse age** (Days; range)	78 (50 - 101)	75 (43 - 96)
**Mouse Body Weight** (Grams; range)	23.5 (17.0 – 27.4)	24.3 (19.3 – 30.8)
**Tumour Latency** (Days; range)	54.2 (20 - 89)	47.0 (33 - 85)
**Tumour Size**		
<10mm	6 (24)	6 (21)
10 – 15mm	9 (36)	10 (36)
16 – 20mm	8 (32)	7 (25)
>20mm	2 (8)	5 (18)
**Tumour Weight** (Milligrams; range)	469.8 (95.1 – 953.9)	449.6 (93.7 – 1366.3)
**Tumour Grade**		
2	4 (16)	2 (7)
3	7 (28)	7 (25)
4	14 (56)	19 (68)

### The effect of ovarian cycle stage on the 21-gene signature in ER-positive mouse mammary tumours

**Fig. 2:**
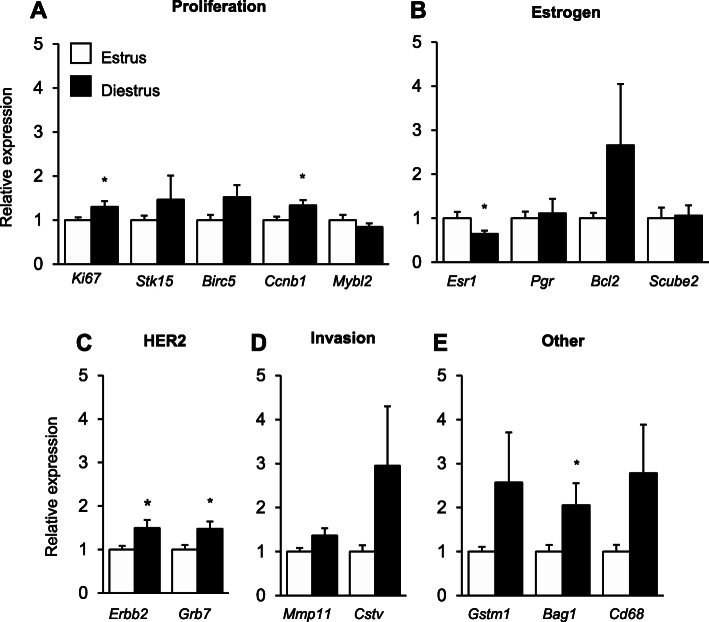
The effect of ovarian cycle stage on the 21-gene signature. Mammary tumours were collected from mice at either the estrus (*n* = 25) or diestrus (*n* = 28) phase of the ovarian cycle. Expression of the genes included in the 21-gene signature were measured by real-time PCR; including (**A**) 5 Proliferation (*Ki67, Stk15, Birc5, Ccnb1, Mybl2*); **B** 4 Estrogen (*Esr1, Pgr, Bcl2, Scube2*); **C** 2 HER2 (*Erbb2, Grb7*); **D** 2 Invasion (*Mmp11, Cstv*); and (E) 3 Other (*Gstm1, Bag1, Cd68*) group genes. Gene expression was normalised to the average of 5 reference genes (*Actb, Gapdh, Rplp0, Gus, Tfrc*). Results are presented relative to tumours collected at estrus. All data are presented as mean + SEM. Statistical significance was determined when *p* ≤ 0.05 using independent t-tests. * signifies *p* ≤ 0.05

To determine whether ovarian cycling affects the 21-gene signature, gene expression was assessed through quantitative real time-PCR and compared between tumours collected at estrus and diestrus phases of the ovarian cycle. Expression of 6 Oncotype DX signature genes were differentially expressed. Tumours collected from mice at diestrus exhibited a significant increase in the expression of genes *Mki67* (*p* = 0.05), *Ccnb1* (*p* = 0.02), *Erbb2* (*p* = 0.03), *Grb7* (*p* = 0.02), and *Bag1* (*p* = 0.05), compared to tumours collected at estrus. Conversely, expression of *Esr1* (*p* = 0.02) was significantly reduced in tumours collected at diestrus compared to estrus (Fig. 2).

**Fig. 3:**
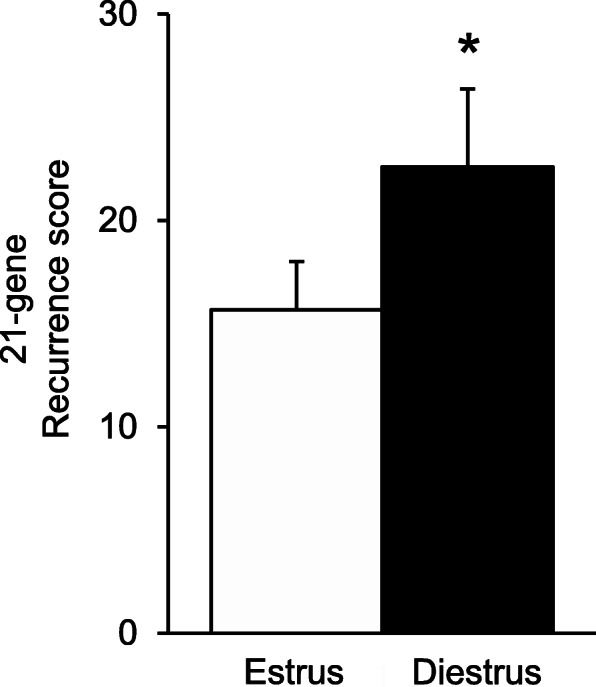
The effect of ovarian cycle stage on 21-gene experimental recurrence scores. Estrogen receptor-positive mammary tumours were collected from *Mmtv-Pymt* transgenic mice at either the estrus (*n* = 25) or diestrus (*n* = 28) phase of the ovarian cycle. 21-gene recurrence scores were calculated from reference-normalised gene expression as described in the methods. Data are presented as mean + SEM. Statistical significance was determined when *p* < 0.05 using Student’s t-test. *signifies *p* < 0.05

We next sought to determine how changes in the 21-gene signature with ovarian cycle stage impact experimental recurrence scores. Using reference-normalised gene expression, 21-gene experimental recurrence scores were calculated for each tumour. This recurrence score is analogous to the Oncotype DX Recurrence Score. Tumours collected from mice at diestrus show a significant increase in experimental recurrence score (21.8 ± 2.4; mean ± SEM) compared to tumours collected at estrus (15.5 ± 1.9; *p* = 0.039; Fig. 3).

### The effect of ovarian cycle stage on clustering analysis of ER-positive mouse mammary tumours

To further investigate how ovarian cycling affects the gene expression profile, t-Distributed Stochastic Neighbour Embedding (t-SNE) analysis was applied to our gene expression data. The t-SNE technique of dimensionality reduction allows for multi-gene datasets to be visualised in a 2-dimentional plot [30]. Consequently, this allowed us to visualise similarities in gene expression profiles between individual tumours.

**Fig. 4:**
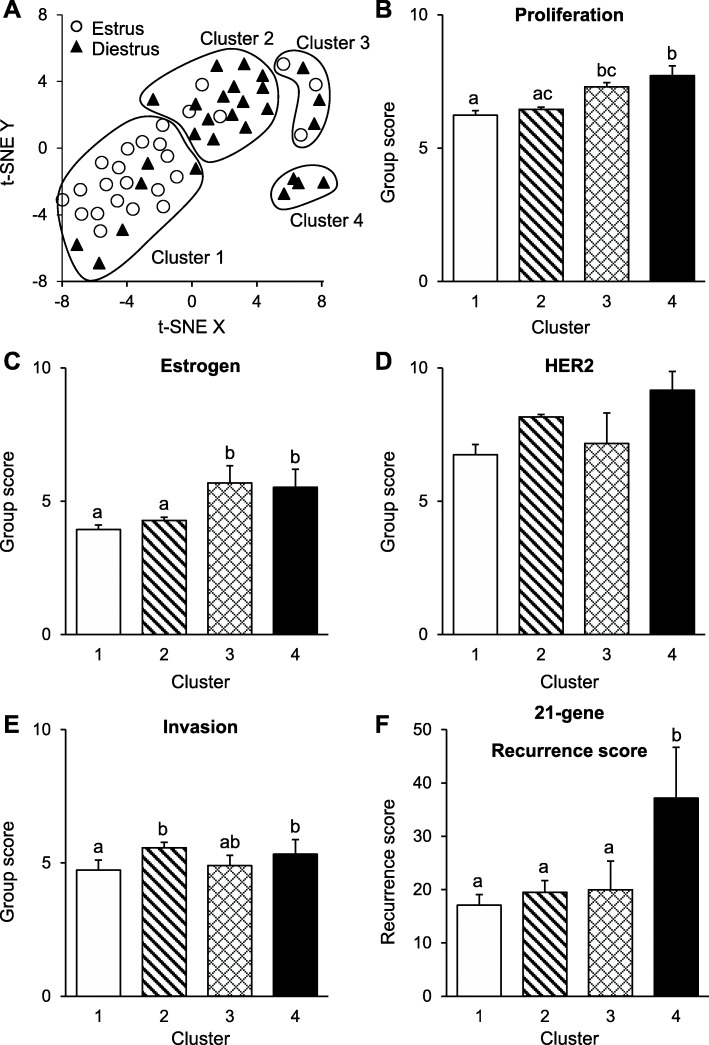
Clustering of mammary tumours based on similarities in gene expression profiles. Mammary tumours were collected from naturally cycling *Mmtv-Pymt* mice at either estrus (*n* = 25) or diestrus (*n* = 28) stages of the ovarian cycle. **A** Mammary tumours were clustered using t-Distributed Stochastic Neighbour Embedding (t-SNE) analysis. Each cluster was characterised by a unique gene expression signature. 21-gene group scores were calculated for each cluster, as described in the methods. Presented are the average group scores for each cluster: **B** Proliferation group, **C** Estrogen group, **D** HER2 group, and **E** Invasion group. **F** The average 21-gene experimental recurrence score for each cluster. All results are presented as mean + SEM. Significance was assessed using one-way analysis of variance with post hoc comparisons performed. Different letters indicate statistical significance (*p* ≤ 0.05) between groups

Tumours grouped into 4 main clusters (Fig. 4A), each which showed distinct gene expression profiles. To highlight the differences in gene expression between clusters, 21-gene group scores were calculated for each cluster (Fig. 4B-E). Clusters 1 and 2 predominantly correspond to tumours collected at estrus and diestrus phases of the ovarian cycle respectively. These clusters exhibited similarities in expression of Estrogen, Proliferation, and HER2 group genes (*p* > 0.05); with the difference between clusters being driven primary by increased expression of Invasion group genes in Cluster 2, compared to Cluster 1 (*p* = 0.005). In respect to the remaining clusters, Cluster 1 and Cluster 2 showed reduced expression of Estrogen group genes, compared to Cluster 3 (*p* < 0.001, *p* = 0.001 respectively) and Cluster 4 (*p* = 0.001, *p* = 0.009 respectively).

We next compared the average recurrence scores for each cluster. Interestingly, tumours in Cluster 4 showed a significant increase in 21-gene recurrence scores (37.1 ± 9.5; mean ± SEM), compared to Cluster 1 (17.1 ± 2.0; *p* = 0.001), Cluster 2 (19.5 ± 2.2; *p* = 0.004), and Cluster 3 (20.0 ± 3.8; *p* = 0.014) (Fig. 4F). No other differences in recurrence scores were observed between clusters (*p* > 0.05). The increased recurrence score of Cluster 4 are being driven by an increased expression of Estrogen, Proliferation, and Invasion group genes, as compared to Cluster 1 (*p* = 0.001, *p* < 0.001, *p* = 0.006 respectively), and Cluster 2 (*p* = 0.009, *p* = 0.002, *p* = 0.25 respectively). No differences in gene expression were observed between Cluster 3 and Cluster 4 (*p* = 0.77, *p* = 0.33, *p* = 0.06 respectively).

### The relative effects of ovarian cycle stage on gene expression versus protein expression

Having found that ovarian cycle stage significantly affects mammary tumour gene expression, we next sought to define how changes in gene biomarker expression translate into changes in protein biomarker expression. In the clinic, the assessment of ER, PR, HER2 and Ki67 protein is the gold standard for informing treatment decisions [1, 3], as their expression predicts the tumours likely response to therapy [31–33]. Therefore, we focused our analysis on assessing concordance between gene and protein expression of ER, PR, HER2 and Ki67.

**Fig. 5:**
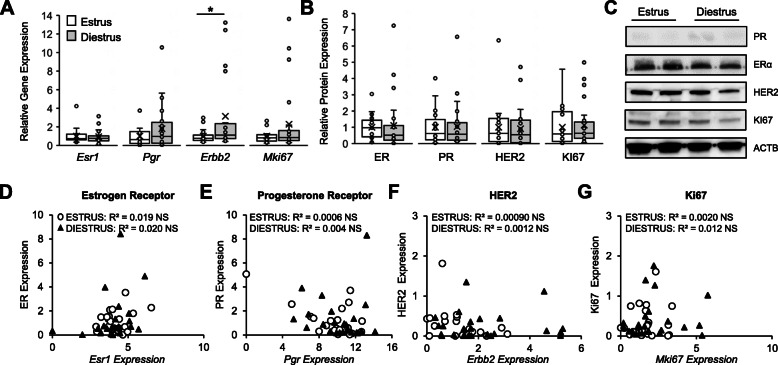
Correlation between gene and protein biomarker expression with ovarian cycle stage. Hormone receptor-positive secondary mammary tumours were collected from *Mmtv*-*Pymt* mice at either estrus (○; *n* = 21) or diestrus (▲; *n* = 24) stages of the ovarian cycle. Gene and protein expression of breast cancer biomarkers, including the estrogen receptor (ER), progesterone receptor (PR), human epidermal growth factor receptor-2 (HER2), and marker of proliferation Ki67 were assessed through (**A**) real-time PCR and (**B**) Western blot analyses. Results are presented relative to β-actin expression, and normalised to estrus. Data are presented as boxplots. Individual values are identified by dots. The mean is identified by an ‘x’. Statistical analysis was conducted using student’s T-test. *signifies *p* < 0.05. **C** Representative Western blots of total ER, PR, HER2, Ki67 and β-actin protein in tumors collected from mice at estrus or diestrus. Correlation between gene and protein expression for (**D**) ER, (**E**) PR, (**F**) HER2, and (**G**) Ki67. Data are presented relative to β-actin expression. Spearman’s correlations for ovarian cycle stage are presented. NS = not significant

Secondary mammary tumours were collected from *Mmtv-Pymt* transgenic mice at either the estrus (*n* = 21) or diestrus (*n* = 24) phase of the ovarian cycle. All tumours were ER and PR-positive, as confirmed by IHC analysis (data not shown). mRNA expression of *Esr1, Pgr, Erbb2,* and *Ki67* was assessed through quantitative real-time PCR. Tumours collected from mice at diestrus showed a significant increase in the expression of *Erbb2* compared to tumours collected from mice at estrus (*p* = 0.03; Fig. 5A). Expression of *Ki67* trended to increase, but did not reach significance (*p* = 0.09). No changes in *Esr1* (*p* = 0.79) or *Pgr* (*p* = 0.17) expression were observed (Fig. 5A).

To assess how ovarian cycle stage affects protein biomarker expression, total ER, PR, HER2 and Ki67 protein was assessed through western blot analysis. No significant differences were observed in the expression of any protein biomarkers between tumours collected at estrus, compared to tumours collected at diestrus (*p* > 0.05; Fig. 5B). Examples of western blots are presented in Fig. 5C and Supplementary figure 5.

To assess whether changes in gene expression with ovarian cycle stage translate into changes in protein expression, we next assessed concordance between gene and protein expression using Spearman’s correlations. We found generally poor correlation between gene expression and protein expression of ER (estrus *r* = 0.019, diestrus *r* = 0.020, Fig. 5D), PR (estrus *r* = 0.0006, diestrus *r* = 0.004, Fig. 5E), HER2 (estrus *r* = 0.00090, diestrus *r* = 0.0012, Fig. 5F), and Ki67 (estrus *r* = 0.0020, diestrus *r* = 0.012, Fig. 5G).

## Discussion

### Ovarian cycling critically affects the 21-gene signature and experimental recurrence scores in ER-positive mouse mammary tumours

The Oncotype DX 21-gene Recurrence Score is predictive of adjuvant chemotherapy benefit for women with early-stage, hormone receptor (HR)-positive, HER2-negative breast cancer and is recommended in clinical guidelines for guiding adjuvant chemotherapy treatment decisions. However, the Recurrence Score algorithm was largely developed in postmenopausal women, and it remains unclear whether it is suitable for use in premenopausal women, where fluctuations in estrogen and progesterone during the menstrual cycle affect gene expression.

This study in mice provides pre-clinical evidence that menstrual cycling might affect Oncotype DX Recurrence Scores for premenopausal women. Tumours dissected from mice at diestrus, which is characterised by high concentrations of progesterone and an intermediate estrogen concentration [28, 34–36], analogous to the luteal phase in humans, show a significant increase in 21-gene recurrence scores, compared to tumours collected at estrus. Increased expression of Proliferation and HER2 group genes largely contributed to the increased 21-gene recurrence score observed in tumours collected at diestrus. Critically, in premenopausal women, proliferation and HER2 gene expression has been shown to fluctuate across the course of the menstrual cycle. Highest proliferative activity of breast cancer samples are observed during the luteal phase, with increasing concentrations of progesterone [14, 37]. Similarly, HER2 expression peaks during the luteal phase [15]. These findings are consistent with a recent study in women [13] which demonstrated that results generated by the Oncotype DX 21-gene algorithm were critically affected by patient age at the time of tissue collection, and that these age-related differences were driven by variable expression of proliferation and HER2 group genes.

Taken together, these data suggest that the Oncotype DX 21-gene assay may be less precise for cycling premenopausal women, compared to non-cycling postmenopausal women, as Recurrence Scores may partially depend on the menstrual cycle stage at the time of tissue collection. For premenopausal women, Oncotype DX testing of hormone responsive tumours may produce a higher Recurrence Score for a tumour sampled during the luteal phase of a woman’s menstrual cycle, when circulating concentrations of progesterone are high, compared to that same breast cancer sampled during the follicular phase, when progesterone is low.

### The effects of ovarian cycle stage on 21-gene experimental recurrence scores are limited to a subset of ER-positive tumours

There is significant inter-tumour and intra-tumour heterogeneity within ER-positive breast tumours, and this contributes to their heterogeneous clinical outcomes, variable response to chemotherapy, and also how these tumours respond to ovarian hormones. Therefore, certain tumours might be more sensitive to fluctuations in circulating concentrations of ovarian hormones during the estrous cycle. Indeed, in mice, we identified a subset of ER-positive tumours collected at diestrus (Cluster 4) that showed a significant increase in 21-gene recurrence scores, compared to the remaining tumour clusters. Tumours in Cluster 4 may be more sensitive to fluctuations in estrogen and progesterone during the ovarian cycle, and consequently, more prone to cycle-induced changes in recurrence scores.

Increased expression of genes downstream of estrogen signalling reflects tumour responsiveness to ovarian hormones. Tumours exhibiting high expression of estrogen-regulated genes are more dependent on hormone signalling for growth, and therefore may be more sensitive to fluctuations in estrogen and progesterone during the ovarian cycle. Indeed, Cluster 4, which showed highest 21-gene recurrence scores, also exhibited highest expression of estrogen-regulated genes, consistent with the possibility that these tumours are more sensitive to hormonal fluctuations. Additionally, this cluster was characterised by increased expression of proliferative genes, which have been previously described as being affected by ovarian cycle stage [17, 18]. We suggest that Cluster 4 is more sensitive to estrogen and progesterone, and an increase in expression of Proliferation-group genes at diestrus is the key driver behind their increased recurrence scores.

Unfortunately, serum concentration of estrogen and progesterone could not be determined in this cohort of mice. Many previous studies have employed a radioimmunoassay (RIA) approach to investigate mouse estradiol and progesterone in serum from naturally cycling mice. The commercial RIA kits DSL-4800 and DSL-3400 (Diagnostic Systems Laboratories, Webster,TX, USA) that measure estradiol and progesterone respectively have been well-validated and are considered the industry standard. Unfortunately, these kits have been discontinued, and none of the currently available ELISA kits are validated for serum hormones in naturally cycling mice, and none that we trialled were found to be specific for estradiol or progesterone (data not shown).

It is possible that for a majority of ER-positive tumours, ovarian cycle stage does not affect recurrence scores. However, the underlying tumour biology that drives an increased sensitivity to cycle-induced changes remains unclear. Furthermore, while we report that the ovarian cycle stage can impact 21-gene experimental recurrence scores, the cycle stage and hormonal environment that reflects the true tumour biology and the likely response from adjuvant chemotherapy is not known.

### The effects of ovarian cycle stage are likely more pronounced on gene expression versus protein expression

Although we found that ovarian cycle stage significantly affects gene biomarker expression in murine mammary tumours, protein biomarker expression was not significantly impacted. It is important to appreciate that reduced mRNA expression does not necessarily lead to reduced expression of the biologically active protein. Indeed, previous studies report that gene expression is more sensitive to external stimuli than its protein counterparts [38], and suggest that changes in gene expression are not necessarily mirrored by changes in protein expression [39, 40].

Discordance between gene and protein expression in *Mmtv-Pymt* mammary tumours may be due to the fluctuations in circulating hormones during the ovarian cycle, and the relative effect of hormonal stimulation on gene versus protein expression. We suggest that gene and protein expression are not directly comparable, and that the effects of ovarian hormones are likely more pronounced on gene expression—and by extension gene expression-based algorithms—compared to traditional protein-based methods. While it is believed that gene expression-based algorithms offer improved treatment decision-making capabilities compared to protein-based methods, the increased sensitivity of gene expression to hormonal stimulation cautions against the adoption of gene-based algorithms for guiding treatment decisions for premenopausal breast cancer patients.

## Conclusion

Ovarian cycle stage in naturally cycling *Mmtv-Pymt* mice significantly affects the 21-gene signature in mammary tumours. Tumours collected from mice at diestrus exhibit a significant increase in Proliferation- and HER2-group gene expression, and increase in 21-gene experimental recurrence scores. For premenopausal women, the menstrual cycle stage the time of tissue collection may affect the Oncotype DX 21-gene Recurrence Score. However, whether Oncotype DX Recurrence Scores in women are similarly affected by menstrual cycle stage is not yet known. To address this question and ensure young breast cancer patients receive optimal treatment advice, future prospective studies using breast cancer samples from premenopausal women with known cycle stage are required.

## Supplementary Information

**Additional file 1.**

## Data Availability

The datasets used and/or analysed during the current study are available from the corresponding author on reasonable request.

## Abbreviations

ER: Estrogen receptor
HER2: Human epidermal growth factor receptor
RS: 21-gene experimental recurrence score
cDNA: Complementary DNA (cDNA)
*ACTB*: Actin Beta
*BAG1*: BCL2-Associated Athanogene
*BCL2*: B-Cell CLL/Lymphoma 2
*BIRC5*: Baculoviral IAP Repeat-Containing 5 (Survivin)
*CCNB1*: Cyclin B1
*CD68*: CD68 Molecule
*CSTV*: Cathepsin V
*ERBB2*: Erb-B2 Receptor Tyrosine Kinase 2
*ESR1*: Estrogen Receptor gene
*GADPH*: Glyceraldehyde-3-Phosphate Dehydrogenase
*GRB7*: Growth Factor Receptor-Bound Protein 7
*GSTM1*: Glutathione S-Transferase Mu 1
*GUSB*: Glucuronidase Beta
*MKI67*: Marker of Proliferation Ki-67
*MMP11*: Matrix Metallopeptidase 11 (Stromelysin 3)
*MYBL2*: Myb Proto-Oncogene Like 2
*PGR*: Progesterone Receptor
*RPLP0*: Ribosomal Protein Lateral Stalk Subunit P0
*SCUBE2*: Signal Peptide, Cub Domain and Egf Like Domain Containing 2
*STK15*: Aurora Kinase A
*TFRC*: Transferrin Receptor
