# Supplementary material for: Ovarian cycle stage critically affects 21-gene recurrence scores in Mmtv-Pymt mouse mammary tumours
Source: BMC Cancer. 2021 Jun 26;21:736. doi: 10.1186/s12885-021-08496-y (PMC8236154; doi:10.1186/s12885-021-08496-y)
Supplement: Supplementary file 1 — Additional file 1. [file 12885_2021_8496_MOESM1_ESM.pdf]

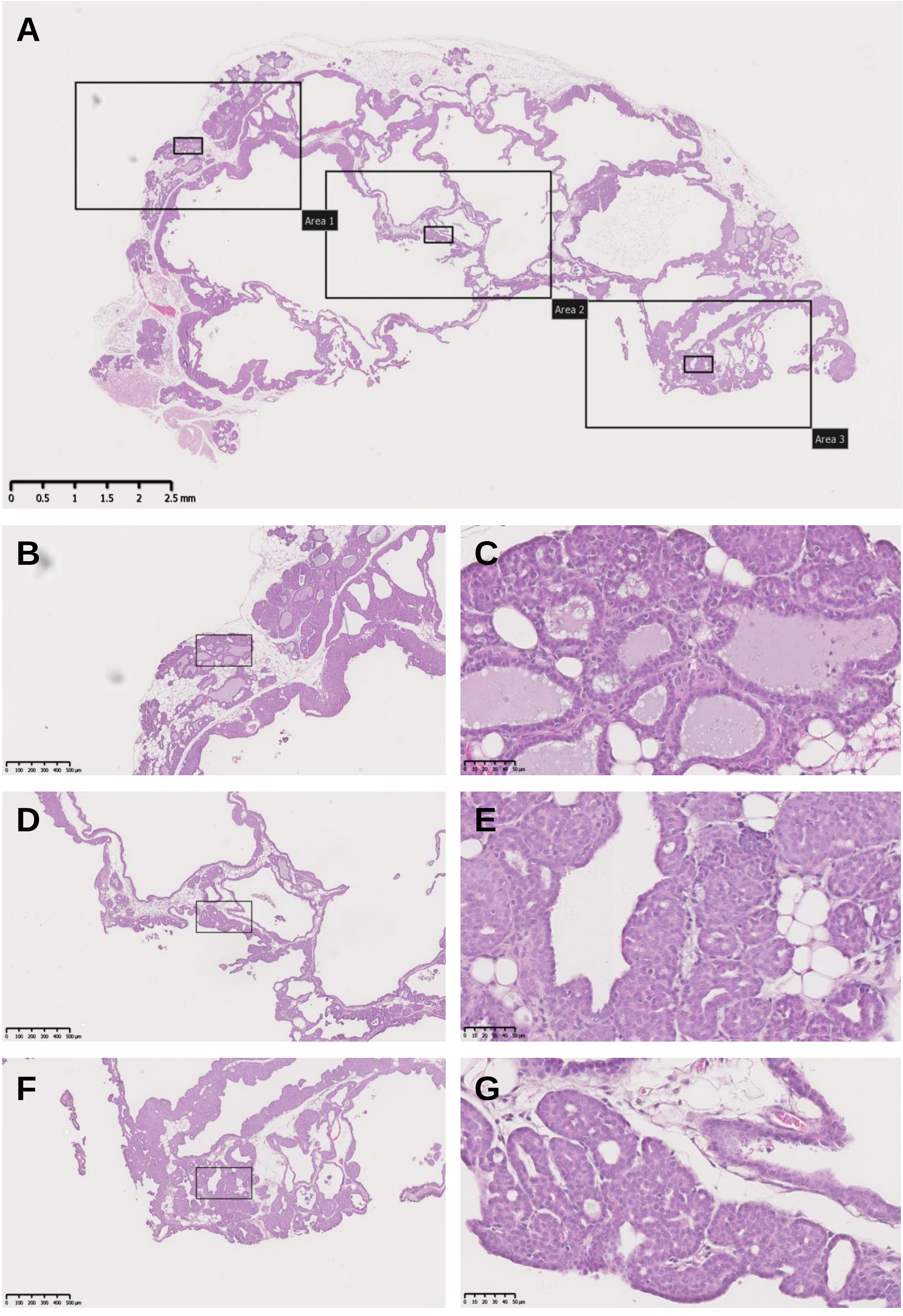

Supplementary Figure 1: Haematoxylin and eosin stain of a grade 2 *Mmtv-Pymt* mammary tumour. Tumours were assessed and graded by a veterinary pathologist. (A) The whole tumour section at 1.25× magnification, with individual areas at higher magnification annotated. Area 1 at (B) 5× and (C) 40× magnification; Area 2 at (D) 5× and (E) 40× magnification; Area 3 at (F) 5× and (G) 40× magnification.

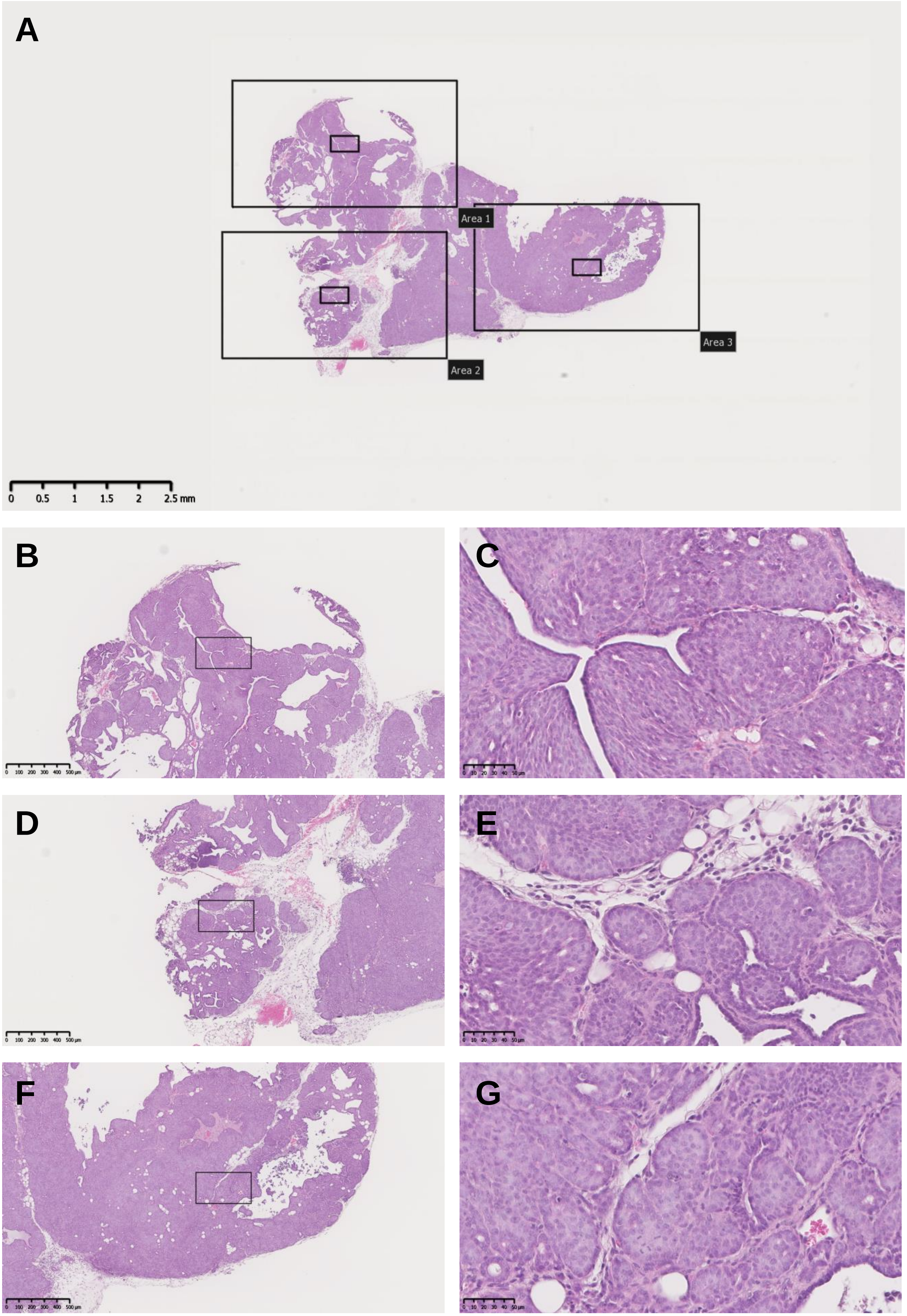

Supplementary Figure 2: Haematoxylin and eosin stain of a grade 3 *Mmtv-Pymt* mammary tumour. Tumours were assessed and graded by a veterinary pathologist. (A) The whole tumour section at 1.25× magnification, with individual areas at higher magnification annotated. Area 1 at (B) 5× and (C) 40× magnification; Area 2 at (D) 5× and (E) 40× magnification; Area 3 at (F) 5× and (G) 40× magnification.

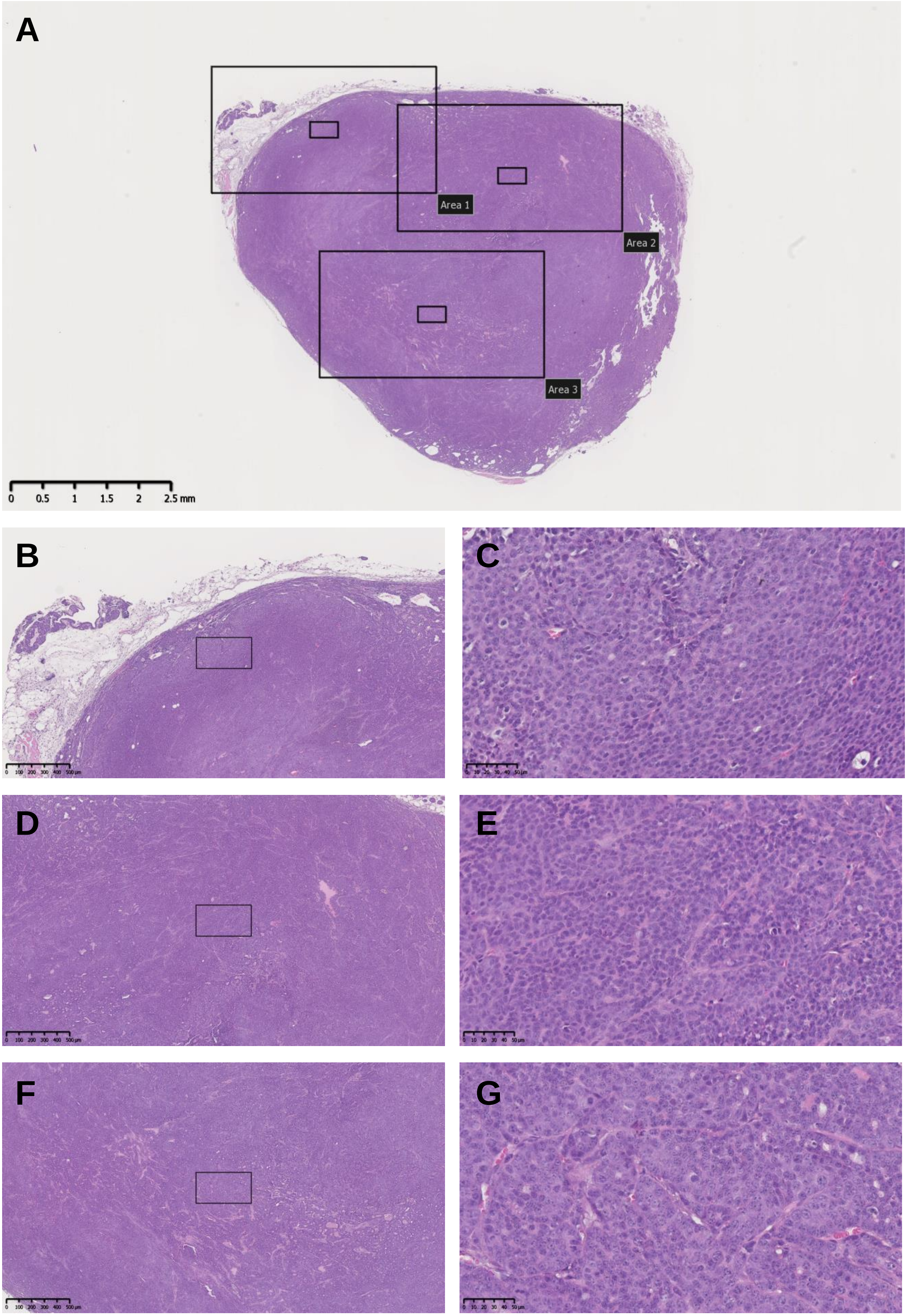

Supplementary Figure 3: Haematoxylin and eosin stain of a grade 4 *Mmtv-Pymt* mammary tumour. Tumours were assessed and graded by a veterinary pathologist. (A) The whole tumour section at 1.25× magnification, with individual areas at higher magnification annotated. Area 1 at (B) 5× and (C) 40× magnification; Area 2 at (D) 5× and (E) 40× magnification; Area 3 at (F) 5× and (G) 40× magnification.

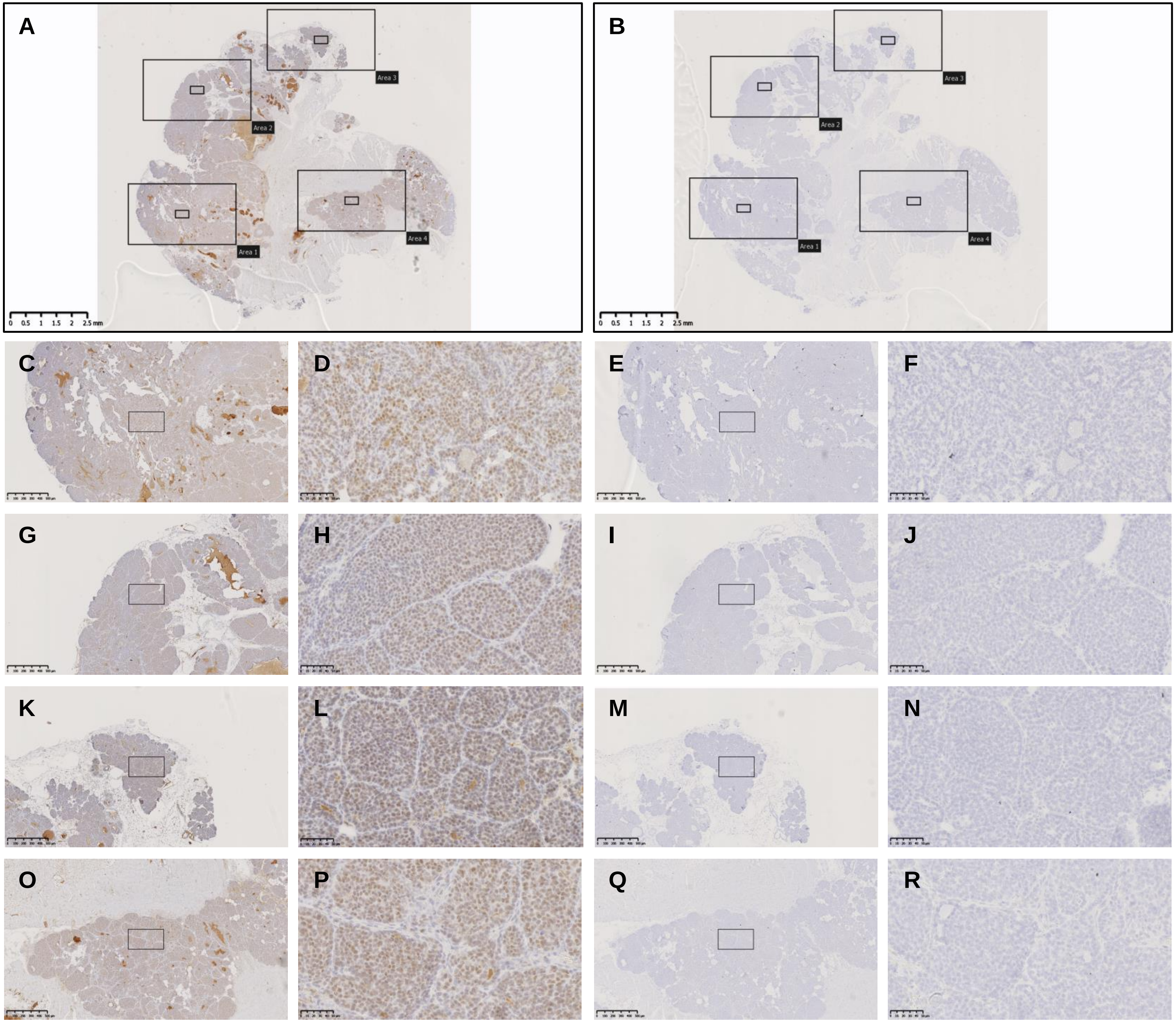
A
Area 1
Area 2
Area 3
Area 4
0 0.5 1 1.5 2 2.5 mm
B
Area 1
Area 2
Area 3
Area 4
0 0.5 1 1.5 2 2.5 mm
C
D
E
F
G
H
I
J
K
L
M
N
O
P
Q
R

Supplementary Figure 4: Estrogen receptor expression in *Mmtv-Pymt* mammary tumours. (A) Whole tumour sections stained for estrogen receptor (ER) expression and (B) secondary antibody only controls. Slides were imaged at 1.25× magnification, with individual areas presented at higher magnification are annotated. ER staining in Area 1 at (C) 5× and (D) 40× magnification; Area 2 at (G) 5× and (H) 40× magnification; Area 3 at (K) 5× and (L) 40× magnification; and Area 4 at (O) 5× and (P) 40× magnification. Secondary antibody only controls for each area are also presented: Area 1 at (E) 5× and (F) 40× magnification; Area 2 at (I) 5× and (J) 40× magnification; Area 3 at (M) 5× and (N) 40× magnification; and Area 4 at (Q) 5× and (R) 40× magnification. Scale bars are presented.

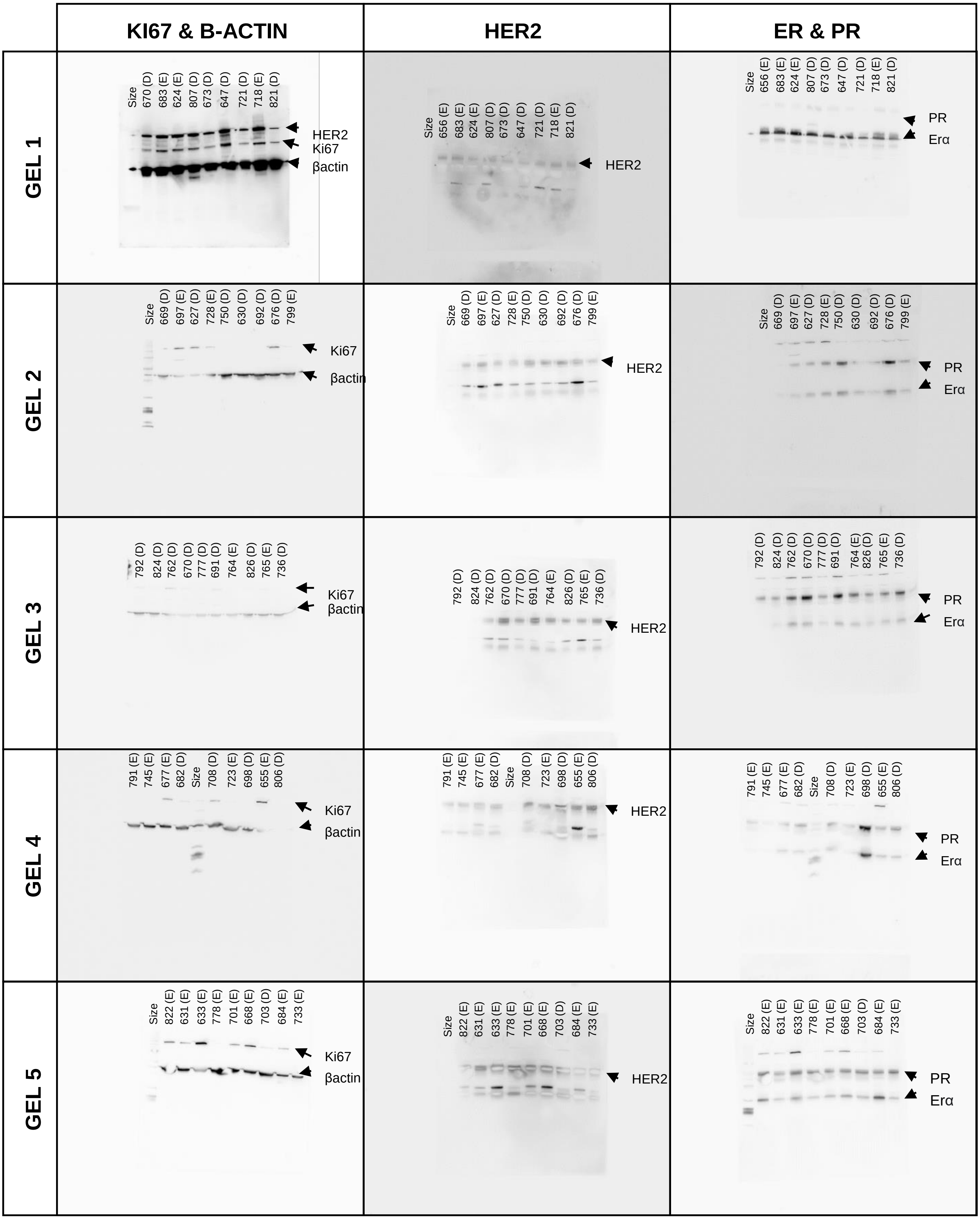

Supplementary Figure 5: Western Blot membranes assessing total Ki67, βactin, HER2, ER, and PR protein in *Mmtv-Pymt* mammary tumours. *Mmtv-Pymt* mammary tumours were collected from mice at either the estrus (E) or diestrus (D) phase of the ovarian cycle**.** Total protein was extracted from tumours using Triton X-100 lysis buffer. A total of 20μg of protein was separated on a 12% polyacrylamide gel at 30mA for 40 minutes, prior to being transferred onto a nitrocellulose membrane. Each lane represents samples obtained from an individual tumour. Membranes were probed with mouse anti-ER and mouse anti-PR primary antibodies, followed by goat anti-mouse secondary antibodies; or rabbit anti-HER2, rabbit anti-Ki67, and rabbit anti-βactin primary antibodies, followed by goat anti-rabbit secondary antibody. Membranes were exposed using enhanced chemiluminescence. A cropped version of these membranes are presented in Figure 5.
